# Task-dependent cold stress during expeditions in Antarctic environments

**DOI:** 10.1080/22423982.2017.1379306

**Published:** 2017-10-08

**Authors:** Drew M. Morris, June J. Pilcher, Robert B. Powell

**Affiliations:** ^a^ Department of Psychology, Clemson University, Clemson, SC, USA; ^b^ Department of Parks, Recreation, and Tourism Management; Clemson University, Clemson, SC, USA

**Keywords:** Occupational health, cold stress, environmental stress, tourism, Antarctica

## Abstract

This study seeks to understand the degree of body cooling, cold perception and physical discomfort during Antarctic tour excursions.

Eight experienced expedition leaders across three Antarctic cruise voyages were monitored during occupational tasks: kayaking, snorkelling and zodiac outings. Subjective cold perception and discomfort were recorded using a thermal comfort assessment and skin temperature was recorded using a portable data logger. Indoor cabin temperature and outdoor temperature with wind velocity were used as measures of environmental stress. Physical activity level and clothing insulation were estimated using previous literature.

Tour leaders experienced a 6°C (2°C wind chill) environment for an average of 6 hours each day. Leaders involved in kayaking reported feeling colder and more uncomfortable than other leaders, but zodiac leaders showed greater skin temperature cooling. Occupational experience did not predict body cooling or cold stress perception.

These findings indicate that occupational cold stress varies by activity and measurement methodology. The current study effectively used objective and subjective measures of cold-stress to identify factors which can contribute to risk in the Antarctic tourism industry. Results suggest that the type of activity may moderate risk of hypothermia, but not discomfort, potentially putting individuals at risk for cognitive related mistakes and cold injuries.

## Introduction

Harsh environmental conditions impact health and task performance through psychophysiological, physiological and environmental mechanisms [1]. Outdoor workspaces yield variable air quality and lighting, uncontrollable noise exposures, modified social protocol and spatial configurations that contribute to environmental stress [2,3]. Among these challenges, thermal stress is often overlooked as a significant occupational stressor. Cold working conditions have been associated with painful musculoskeletal disorders [4], loss of manual strength and dexterity [5], increased occurrence of slips, trips and falls [6] and a variety of cognitive impairments which increase the risk of an accident [7]. These cognitive impairments can include limited attention, curtailed memory and reasoning ability and impulsiveness; even at moderate levels of stress [8,9]. Moreover, risk of cold-related impairment also increases with duration and intensity of exposure, highlighting the hazard of performing critical tasks in consistently cold environments [8].

**Figure 1.**
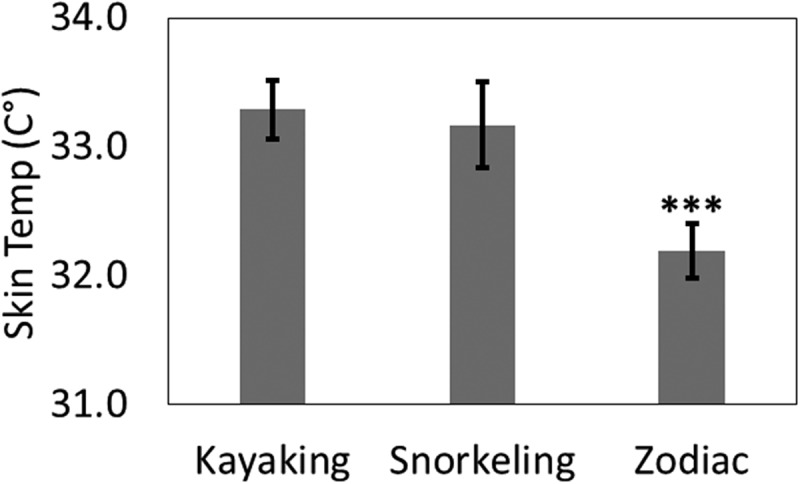
Skin temperature means by task (error bars from standard error). *** p <.001.

**Figure 2.**
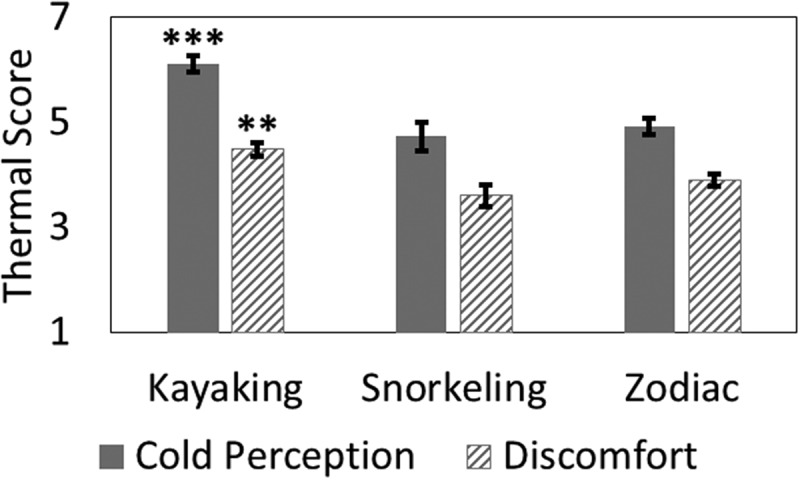
Thermal comfort assessment score means by task (error bars from standard error). Cold Perception (1 = Very Hot, 9 = Very Cold), Discomfort (1 = Very Comfortable, 5 = Very Uncomfortable). *** p<.001, ** p<.01.

Among the occupations that may be at high risk for thermal stress hazards are those near the Polar Regions, particularly the tourism industry [10]. The Antarctic continent is one of the most physiologically challenging environments on Earth and, yet, the number of ship-borne tourists in Antarctica has increased by over 300% in the last decade to include tens of thousands of visitors [11]. Given the development of tourism in the polar region, there is an increased need for research on health and safety risks related to polar environments [12]. Indeed, human performance and health near the Polar Regions has been a popular topic for environmental psychology and industrial medicine for several decades [13–15]. While occupational research surrounding cold stress impairment in the mining industry or cold-related hazards during military exercises have been thoroughly explored, research on environmental stress within tourism is exceedingly limited [16,17].

While the interaction between environmental stress and task performance is important in any industry, environmental stress may be especially high in polar tourism [18,19]. Expedition leaders in particular are responsible for equipment management, passenger safety and experiential programming (location, interpretation and education) and, yet, also experience the most detrimental environmental exposure [20]. Under wet and freezing conditions, leaders are the first individuals to go outdoors and the last to come indoors, the first to become wet and the last to dry off, endure the most wind chill and spend the most time outdoors during the cooler parts of the day (i.e. mornings and evenings) [21,22]. Moreover, these environmentally stressful expeditions may last for up to 5-hours and offer little to no shelter from the elements during that time, compounding stress with duration [23]. As a result, the individuals with the most responsibility are also the most likely to have attentional and decision-making impairments. Indeed, researchers have routinely shown how cold water immersion can limit attention and decision-making, even without affecting core temperature [24,25]. These attentional and decision-making impairments can make it difficult to keep track of members on an outing, make the best judgement following an unexpected event or simply recall procedure.

Although the impact of cold environments on task performance is well documented, the degree to which cold stress occurs in the tourism industry is unknown. Literature in other maritime industries suggests cold stress should be a serious concern, but unique factors limit the generalizability of these findings [26]. Although cold temperatures contribute to cold stress, factors such as clothing insulation, thermogenesis from movement, personal experience, cold perception and subjective discomfort have all been shown to moderate the thermal stress/performance relationship [27]. As such, an applied study is needed to assess how these variables and other underlying environmental factors such as wind chill are contributing to risk [28].

The purpose of the current study is to explore the degree to which body cooling, cold perception and subjective discomfort occur in polar environments. In particular, this study focuses on expedition leaders and discriminates between different types of tasks and activities associated with their occupation. The authors hypothesise that the type of tasks engaged in will moderate physiological and perceived cooling. First, the authors hypothesise that skin temperature will be higher during more physically active excursions. Second, the authors hypothesise that cold perception and subjective discomfort will be lower during more physically active excursions.

## Method

### Sample

Eight expedition leaders (five males and three females, age 40.37±10.23 years) who were associated with a single Antarctic cruise ship company participated in this study. Participants had 6.8 (±7.21) years of experience in the Antarctic tourism industry and lead either kayaking, snorkelling or zodiac/walking outings during the cruise ship voyages. All participants self-identified as having good-to-excellent mental and physical health. The study was approved by the university institutional review board and all participants provided signed consent prior to participation. All procedures were in accordance with the ethical standards of the 1964 Helsinki declaration and its later amendments or comparable ethical standards.

Data collection took place across three separate cruise ship voyages of 9–11 days each. During each voyage, an expedition leader would lead a group of passengers on between 10 and 12 outings (excursions). Each excursion lasted between 1 and 5 hours. Two participants were present on three voyages, four participants were present on two voyages and two participants were present on one voyage. Clothing varied between tasks and leaders, but consisted of an under-layer (e.g. long underwear), middle-layer (e.g. wool leggings) and an outer later (e.g. water/wind proofing layer); offering approximately 2.00–3.00 Clo of body insulation. Snorkelling and kayaking leaders wore a dry suit for their outer layer, while zodiac drivers wore a thinner waterproofing layer.

### Materials

#### Participant measures

Each participant wore two pieces of equipment during each excursion to objectively monitor activity and skin temperature. Skin temperature was recorded using a small (< 1 oz) button-sized temperature data logger (SmartButton, ACR Systems Inc., Surrey, B.C., Canada). The sensor was attached to the top of the forearm using breathable medical tape and logged temperature at a rate of 0.1 Hz. Activity level during an excursion was recorded using a small (< 1 oz) actigraphic logger (Actiwatch, Mini Mitter Company Inc., Bend, OR) [29]. The sensor was attached to a fabric lanyard and worn around the neck in-between the under-layer and middle-layer of clothing and logged movement on an electronic triaxis accelerometer once every 2 minutes. All equipment was pre-programmed to log data continuously through the voyage. Participants were only required to wear the physiological equipment during excursions. A temperature and relative humidity data logger (TRH 1000, ACR Systems Inc., Surrey, B.C., Canada) remained on the ship to record environmental conditions during the excursions. Subjective feelings of cold were recorded on a standard two-question thermal comfort assessment scale (TCA); perceived thermal stress (1 = Very Hot, 9 = Very Cold) and thermal comfort (1 = Very Comfortable, 5 = Very Uncomfortable) [9]. Each participant completed the TCA before and immediately after each excursion.

#### Tasks

The kayaking task involved the kayaking leaders preparing the equipment for the tourists on the back deck of the ship. The leaders then took passengers on a sea kayaking trip, depending on weather conditions and passenger motivation. After the excursion, the leader was then responsible for storing the equipment until the next excursion. This task was considered a high-activity task. Similarly, the snorkelling task involved the snorkelling leaders preparing the equipment for the tourists. The leaders then took passengers on an open-water snorkelling trip and were responsible for storing the equipment until the next excursion. This task was also considered a high-activity task. The snorkelling task took place in water as opposed to floating on water (approximately 0°C). Similar to the other tasks, the zodiac/walking task involved leaders preparing a zodiac (light powerboat) for the tourists. The leader then took passengers on a boat sightseeing tour, as well as to shore to perform a short distance walking tour. This task was considered a low-activity task.

### Procedure

Participants were contacted and recruited before the cruise ship left port on the first day of each voyage. Upon approval, a general questionnaire was administered to assess job experience and personal medical history. At this same time, the participants were given the movement logger, the skin temperature data logger, a copy of the TCA questionnaire and a log sheet to record responses. Participants were given a demonstration on how and when to wear the equipment and were also given printed instructions with illustrations on how to fill out the data sheet and use the equipment.

Participants logged the time at which each excursion began. Immediately after returning to the ship from the outing, participants completed the TCA questionnaire and logged the time the outing ended. All outings took place simultaneously, leaving and returning to the ship at roughly the same time. However, the time and duration of each outing would vary between days, depending on the weather (e.g. a 09:00–13:00 excursion on Monday then a 07:30–09:00 excursion on Tuesday) and location. Participants continued this protocol approximately twice a day for each day of a voyage. After the ship returned to port, the equipment was collected.

### Data analysis

Data analysis was performed using the IBM SPSS statistical program (SPSS 22; SPSS Inc., Chicago, IL). Data analysis treated each outing of each participant of each voyage as a separate case. This allowed for 156 recorded excursions across the whole study; 57 kayaking excursions, 23 snorkelling excursions and 76 zodiac/walking excursions. Wind chill was calculated using the National Weather Service’s new wind chill equivalent temperature formula [30]. Skin temperature and activity level were averaged within each outing to get a mean value for a single excursion. Severe movement noise was present in the zodiac excursion activity data, due to the movement of the boat in the water. As a result, actigraphic activity data could only be analysed for the kayaking and snorkelling leaders. The zodiac excursion activity level was then estimated using an energy expenditure assessment and categorised as low activity compared to snorkelling and kayaking [31]. This low-activity categorisation is primarily due to the task being performed in a stationary position. A bivariate correlation was used to show the relationship between duration of excursion and temperature of excursion. Three independent samples *t*-tests with Bonferroni correction for family-wise error were used to test for mean differences between high and low experience expedition leaders to account for possible physiological and psychological acclimation related to experience. A 3-level independent samples ACNOVA with a Bonferroni post-hoc analysis was used to test for mean differences between the three excursion tasks. Because of the study design, a subject covariate was used to account for individual differences.

## Results

Average duration of excursions, as well as indoor cabin temperature and outdoor temperature remained similar between the three voyages (Table 1). Variations in wind speed contributed to differences in average wind chill for each voyage. While on the ship between excursions, indoor cabin temperature ranged between 18° and 24°C. The result of a bivariate correlation showed that the duration of the excursions were negatively correlated with outdoor temperature (r(149)=.20, p=.011). The result of an independent samples *t*–test between excursion leaders with low experience (=2 years, n=4) and those with high experience (>2 years, n=4) in the Antarctica tourism industry found no effect of experience on thermal perception (low 4.85±1.67; high 5.47±1.26), comfort (low 3.64±1.15; high 4.18±.99) or skin temperature (low 32.55°±1.20; high 32.92°±0.97) after excursions, p>.05. Therefore, high and low experience leaders were analysed together.

**Table 1.** Environmental parameters during voyages.

	Voyage					
	1		2		3	
Measure	Mean	SD	Mean	SD	Mean	SD
Indoor cabin temp (°C)	20.97	1.1	20.83	0.9	21.05	1.7
Outdoor temp (°C)	6.19	3.4	6.13	2.1	6.10	1.9
Expedition length (h)	3.29	0.7	3.12	0.8	3.08	0.7
Wind velocity (km/h)	26.36	13.3	12.73	7.9	18.52	13.0
Wind chill (°C)	1.92	5.55	3.50	3.08	2.63	0.94

The result of an independent samples ANCOVA with post-hoc analysis showed that participant skin temperature was significantly colder during zodiac excursions (32.2°±1.8) compared to kayaking (33.3°±1.7) or snorkelling (33.2°±1.6) excursions (F[3,152]=16.41, p<.001; Figure 1). However, the participants perceived the kayaking excursions as feeling significantly colder (F[3,157]=11.07, p<.001) and less comfortable (F[3,157]=5.43, p=.001) than the snorkelling or zodiac excursions (Figure 2). As measured by the activity monitor, there was no significant difference in activity between the two high-activity tasks, kayaking and snorkelling, p>.05.

### Discussion

This study examined the degree to which body cooling, cold perception and discomfort occur in polar environments. The current findings indicate that cold stress during an Antarctic expedition varies by task and measurement methodology, but not by experience. Consistent comfortable cabin temperatures suggest that cold stress was only present during excursions and was not compounded by time spent resting between excursions. In support of the first hypothesis, there were significant differences in body cooling related to the excursion task. Zodiac expedition leaders had significantly lower skin temperature than other expedition leaders during the outing. Because of protective clothing, this lowered skin temperature is likely the result of physiological vasoconstriction in response to temperature loss and not the result of direct contact with the outside air [32]. It is plausible that this disparity in heat loss is due to the wind chill and wetness factors associated with being sedentary when riding in a motorised boat [28]. With an average temperature of 6°C and an average daily wind speed of 10 mph, a modest boat speed of 10 mph modifies wind chill temperatures to below 0°C [30]. In addition, compared to kayaking and snorkelling leaders, zodiac leaders were mostly stationary, resulting in less body heat production.

However, contrary to the second hypothesis, zodiac trip leaders did not report feeling the coldest or the most discomfort in response to the cold. This may be due to powerboat driving being an attentionally-demanding task when the water is littered with ice and other obstacles. Indeed, attentionally-demanding tasks have been shown to dull uncomfortable thermal perception [33]. This suggests that power boat activities may put leaders at increased risk for cold-related injuries, due to an unawareness of cooling. Results also showed that kayaking expedition leaders reported feeling significantly colder and more uncomfortable than other expedition leaders. This is perhaps a function of the difficulty with dressing for high activity levels during kayaking that are interspersed with low-activity occasions (resting) as well as activities such as loading kayaks onto the ship that expose leaders to high levels of evaporative cooling and wind chill while clothes are extremely wet at the end of an excursion [34]. These findings suggest that power boat activities may put leaders at increased risk for cold-related injuries, due to an unawareness of cooling.

Research has shown that exposure to temperatures under 10°C for durations greater than 2 hours can have a significant negative impact on physical and cognitive performance, as well as general health and comfort [8]. As was observed, all expeditions took place in <10°C temperatures and tended to last for more than 3 hours without a break. When considering wind chill effects and wetness due to water splashing or perspiration, these temperatures can become extreme [35]. Additionally, as shown by a negative correlation, longer expeditions were more common on colder days, furthering the risk of cold stress and fatigue [36]. This correlation may be the result of less cloud cover, leading to more appealing skies, with the trade-off of cooler temperatures. Studies have suggested that acclimation to cold environments can moderate response to cold stress, but prior experience was not indicative of subjective or physiological cold tolerance [32,37]. These findings should be considered when addressing occupational health policies within the tourism industry [23].

Leaders reported discomfort and cold perception to varying degrees, but did not show extensive body cooling (<2°C). Experience has shown that equipment failure can result in hypothermia during excursions, but simple discomfort from body cooling is far more common with proper equipment upkeep [38–40]. However, it should still be noted that uncomfortable body cooling has been shown to alter the perception of risk and even increase hazardous decision-making independent of hypothermia [41,42]. Indeed, individuals under discomforting stress have been shown to discount risks associated with unlikely events [41]. This discomfort may explain the relationship seen between increases in occupational accident frequency and decreases in air temperature [43]. Another factor that may play a role in accident occurrence and risk-taking behaviour during cold stress is self-control. The cold presser tests, which uses cold water to uncomfortably cool a localised area of the body, has been routinely used to induce behavioural impulsiveness and limit self-regulation [44]. Applied research has also shown this effect and demonstrated that cold stress leads to more impatient behaviour following a brief cooling period [9]. As such, tour leaders under cold stress that are responsible for the safety of tourists may have impaired risk perception and self-control.

The current study had several limitations which should be addressed in future research. This study did not use any direct measures of workload or attention. Future studies should explore task engagement and attentional requirements to better understand this effect and generate alternative explanations. Because of the uniqueness of the population, the sample size for this study is relatively small. Future research should attempt to sample a larger number of leaders, additional excursion activities and tourists. Although the present study did not see a difference in activity level between high-activity groups, other methods of measuring physical activity may better explain the relationship between movement and cold stress during excursions. Lastly, future research should explore other variables related to occupational safety in polar environments. Other variables of interest include risk-taking probability and risk perception, decision-making and judgement, regulation compliance, pre-existing health risks, physiological measures of workload, as well as occurrences of accidents and injuries (if any).

## Conclusion

Since the early years of occupational health research and polar exploration, health practitioners have explored cold stress and human performance [13]. The current study is one of the first to assess environmental stress within the Antarctic tourism industry. Understanding risks associated with working in such an environment needs to remain a priority as the industry continues to grow in popularity [19]. Currently, protective equipment and safety procedures are in place to minimise risk, but literature is missing objective and subjective measures of cold exposure to better understand the degree to which cold-stress risk persists. Results suggest that active leaders and tourists may not be at risk for hypothermia, but do experience uncomfortable cold stress which may put them at risk for inattention-related mistakes and injuries. In addition, findings show that less active leaders may not perceive how much their body is actually cooling, putting them at risk for injury in the event of excessive wind chill. The current study takes an important first step toward understanding the cold stress risks tour leaders and tourists alike are exposed to and proposes a need for future research in the interest of a safe and sustainable Antarctic tourism industry, and policy development.
